# Berberine Reduces Neurotoxicity Related to Nonalcoholic Steatohepatitis in Rats

**DOI:** 10.1155/2015/361847

**Published:** 2015-10-20

**Authors:** Doaa A. Ghareeb, Sofia Khalil, Hani S. Hafez, Jürgen Bajorath, Hany E. A. Ahmed, Eman Sarhan, Eiman Elwakeel, Maha A. El-Demellawy

**Affiliations:** ^1^Biochemistry Department, Faculty of Science, Alexandria University, Alexandria 21511, Egypt; ^2^Biotechnology Department, Zoology, Faculty of Science, Suez University, Suez, Egypt; ^3^Department of Life Science Informatics, B-IT, LIMES Program Unit Chemical Biology and Medicinal Chemistry, Rheinische Friedrich-Wilhelms-Universität Bonn, Dahlmannstraße 2, 53113 Bonn, Germany; ^4^Pharmaceutical Organic Chemistry Department, Faculty of Pharmacy, Al-Azhar University, Nasr City, Cairo, Egypt; ^5^Pharmacognosy and Pharmaceutical Chemistry Department, Pharmacy College, Taibah University, Al-Madinah Al-Munawarah, Saudi Arabia; ^6^Protein Research Department, Genetic Engineering & Biotechnology Research Institute, City for Scientific Research & Technology Applications, Alexandria, Egypt; ^7^Zewail City of Science and Technology, Helmy Institute for Medical Sciences, Center for Aging and Associated Diseases, Sheikh Zayed District, 6th of October City, Giza 12588, Egypt; ^8^Medical Biotechnology Department, Genetic Engineering & Biotechnology Research Institute, City for Scientific Research & Technology Applications, Alexandria, Egypt

## Abstract

Berberine is a plant alkaloid that has several pharmacological effects such as antioxidant, antilipidemic, and anti-inflammatory effects. Nonalcoholic steatohepatitis (NASH) triggers different aspects of disorders such as impaired endogenous lipid metabolism, hypercholesterolemia, oxidative stress, and neurotoxicity. In this study, we examined the mechanism by which NASH induces neurotoxicity and the protective effect of berberine against both NASH and its associated neurotoxicity. NASH induced rats showed significant impairments in lipid metabolism with increased serum triglycerides, cholesterol, and low-density lipoprotein (LDL). The NASH induced group also demonstrated a significant oxidative stress which is characterized by increased TBARs level and decreased antioxidant capacity such as GSH and SOD levels. Moreover, the NASH induction was associated with inflammation which was demonstrated by increased TNF*α* and nitric oxide levels. Hyperglycemia and hyperinsulinemia were observed in the NASH induced group. Also, our results showed a significant increase in the expression of the acetylcholine esterase (AChE) and amyloid beta precursor protein (A*β*PP). These changes were significantly correlated with decreased insulin degrading enzyme (IDE) and beta-amyloid_40_ (A*β*_40_) and increased beta-amyloid_42_ (A*β*_42_) in the hippocampal region. Daily administration of berberine (50 mg/kg) for three weeks ameliorated oxidative stress, inflammation, hyperlipidemia, hyperglycemia, hyperinsulinemia, and the observed neurotoxicity.

## 1. Introduction

Nonalcoholic fatty liver disease (NAFLD) is the nonphysiological accumulation of fats in the liver due to impaired fatty acids' metabolism. This disease is followed by hepatic injury mediated by inflammatory cytokines, oxidative stress, and mitochondrial dysfunction which promote inflammatory infiltration and fibrosis resulting in steatohepatitis (NASH) [1]. Cholesterol rich environment directs the amyloid precursor protein (APP) to be predominantly cleaved with beta secretase 1 (BACE1), generating the neurotoxic A*β* fragment with manifestations of AD pathology [2]. Furthermore, the decline in cholinergic system markers such as choline acetyltransferase and acetylcholinesterase has been correlated with both the degree of dementia and the number of neuritic plaques. It was shown that hypercholesterolemia increased the risk of Alzheimer disease (AD) [3].

Our previous studies have revealed the role hypercholesterolemia and lipid peroxidation associated with dyslipidemia and the role of insulin resistance in brain tissues and body fluids, implicating their effects in triggering brain damage through the upregulation of acetylcholinesterase [4]. The most effective way to control cholesterol synthesis is inhibiting the 3-hydroxy-3-methylglutaryl coenzyme A reductase (HMGR, EC 1.1.1.88). HMG-CoA reductase catalyzes the conversion of HMG-CoA into mevalonate (MVA). Epidemiological studies showed a potential link between cholesterol-lowering compounds, such as statins, and decreased prevalence or incidence of dementia through targeting lipid metabolizing enzymes [5]. Neither the potential efficacy of statins in treating the dementia nor the mechanisms of the reported statin-induced neuroprotection are well-understood. Also, it was shown that acetyl cholinesterase inhibitors are associated with a range of side effects [6]. It was suggested that insulin resistance may contribute to amyloidosis by interfering with insulin degrading enzyme (IDE) mediated degradation of amyloid A*β* peptides [7].

Berberine is a plant alkaloid with a long history of medicinal use in both Ayurvedic and Chinese culture [8]. Clinical studies showed that the administration of 500 mg berberine per day for 4 weeks reduced LDL-c level by 20% [9], which was mediated by increasing LDLR expression at the posttranscriptional level through stabilization of LDLR mRNA in an extracellular signal-regulated kinase-dependent manner, a mechanism distinct from that of statins [10]. It was shown that berberine has antilipidemic effect in mice [11]. 200–400 mg/kg berberine daily for six weeks with twice weekly injections of CCL_4_ demonstrated hepatoprotective effects in regard to serum liver enzymes and histological examination [12]. Furthermore, berberine has been found to have an inhibitory potential activity against acetylcholinesterase (AChE) and butyrylcholinesterase (BChE) [13].* In vitro*, berberine reduced *β*-amyloid levels (APP_NL_-H4 cells) and downregulate *β*-secretase in HEK293 cells [14]. In our previous studies, berberine extracts were found to inhibit AChE in a competitive manner with inhibition percent value of 50%. Thus, berberine extract is considered as competitive inhibitor to AChE [4, 15].

In this study, we will investigate the mechanism by which nonalcoholic steatohepatitis (NASH) affects liver and consequently develops neurotoxicity. In addition, we will introduce extensive biochemical and computational analyses for the effect of berberine as promising treatment NASH and its associated neurotoxicity.

## 2. Materials and Methods

### 2.1. Chemicals and Reagents

Carbon tetrachloride (CCL_4_), NAD^+^, dimethylaminobenzaldehyde reagent, thiobarbituric acid (TBA), cumene H_2_O_2_, reduced glutathione (GSH), Ellman's reagent (5,5′-dithiobis-(2-nitrobenzoic acid) or DTNB), p-hydroxydiphenyl, trichloroacetic acid, Folin's reagent (sodium 1,2-naphthoquinone-4-sulfonate), acetylthiocholine iodide (ACTI), and berberine chloride were purchased from Sigma Chemical Company (St. Louis, Mo, USA). All the other reagents and commercial kits were purchased from Diamond diagnostic and Biodiagnostic companies (Egypt).

### 2.2. Animals and Experimental Design

Forty adult female Sprague-Dawley rats (weighed from 150–200 g) were procured from the animal house of Faculty of Medicine, Alexandria University, Egypt. Experiments were performed following international ethical standards and according to the Guide for the Care and Use of Laboratory Animals of the National Institutes of Health (Institute of Laboratory Animal Resources 1996). The rats were kept in cages in groups of ten and exposed to approximately 23–25°C with a 12-h light/dark cycle. Food and water were available* ad libitum* for one week (acclimatization period). CCL_4_ was mixed at a concentration of 1.6% (V/V) in olive oil for administration according to standard protocol [16].

The animal groups were classified as follows: Group 1 (control) rats of this group were injected with water (1.2 mL/Kg) three times weekly for 3 weeks then orally administrated 0.5 mL of 20% PEG (polyethylene glycol) for another three weeks as vehicle with free access to tap water. Group 2 (control berberine) was orally received 0.5 mL of 50 mg/kg berberine dissolved in 20% PEG for 3 weeks. Twenty rats were intraperitoneally injected with CCL_4_ solution at a dose of (50 *μ*L/Kg) three times per weeks for three weeks. After the induction period, the rats were subgrouped into 2 groups (10 rats each) as follows; one group was received 0.5 mL PEG 20% as NASH induced group and the other group was received 0.5 mL of 50 mg/kg berberine for another three weeks as the fourth group.

Rats were sacrificed after anesthesia with diethylether inhalation, and the brains were rapidly removed and cut sagittally into left and right hemispheres on an ice-cooled board. The hippocampus was dissected from the left and right hemisphere and stored at −80°C for biochemical tests, sandwich enzyme-linked immunosorbent assay (ELISA), and RNA extraction. Liver tissue was collected and stored in formalin for histological studies. Blood was collected and sera were separated to measure the liver function, lipids profile, *β*-amyloid_40_, prooxidants/antioxidants, and acetylcholinesterase (AChE) measurements.

Brain tissue was weighed and homogenized directly in 9 volumes of cold phosphate buffer using Potter-Elvehjem type glass-Teflon homogenizer. To separate the nuclear debris, the tissue homogenates were centrifuged at 3000 rpm for 15 min at 4°C. Brain supernatant was used for further analysis. For ELISA assays, tissues were homogenized in radioimmunoprecipitation assay buffer containing protease and phosphatase inhibitors [17].

### 2.3. Biochemical Parameters Assays

#### 2.3.1. Determination of Blood and Brain Lipid Peroxidation

The malondialdehyde content, a measure of lipid peroxidation, was assayed in the form of thiobarbituric acid-reactive substances (TBARS) by the method described by Wills [18].

#### 2.3.2. Determination of Blood and Brain Endogenous Antioxidant Activities

(1) *Glutathione peroxidase (Gpx)* activity was calculated according to Paglia and Valentine with the following equation: GPx activity (U/g wet tissue) = *A* × 6.2 × 100/13.1 × 0.05 × 10 [19].
(2) *Reduced glutathione (GSH)* activity was assayed by the method of Jollow et al. and the resulting color was measured immediately at 412 nm [20]. Standard curve was constructed using standard GSH.
(3) *Superoxide dismutase (SOD)* activity was assessed according to method of S. Marklund and G. Marklund [21]. The inhibition percent was calculated according to the following equation: the percentage inhibition = [100 − (*A*_*t*_ min^−1^ mL^−1^ sample)/(*A*_*r*_ min^−1^ mL^−1^ reference)] × 100.

#### 2.3.3. Determination of Serum Triglycerides

Triglyceride was measured by the method described in the commercial triglycerides kit purchased from Biodiagnostic, Egypt [22]. 

*Estimation of Brain and Serum Cholesterol*. The assay was carried out according to the method of Watson using a commercial cholesterol kit purchased from Biodiagnostic, Egypt [23].

#### 2.3.4. Estimation of Low and High Density Lipoproteins (LDL and HDL)

LDL and HDL were assayed according to Friedewald et al. and Lopes-Virella et al., respectively, using the commercial kit [24, 25]. While VLDL was calculated by using the following equation:

(1) $$\begin{array}{c} \text{VLDL}\left(\;\text{mg}/\text{dL}\;\right)=\frac{\text{Blood triglyceride}}{5}. \end{array}$$

#### 2.3.5. Determination of Serum AST and ALT Enzymes

Aspartate aminotransferase (AST) and alanine aminotransferase (ALT) were assayed by the method described in the commercial ALT kit purchased from Biodiagnostic, Egypt [26].

#### 2.3.6. Sandwich ELISA for Quantification of Beta Amyloid 1-40 and 1-42

Rat amyloid beta peptide 1-40 and 1-42 (A*β* 1-40 and A*β* 1-42) were assayed by the method described in the commercial A*β* 1-40 and A*β* 1-42 ELISA kit purchased from Cusabio Biotech Co., China. The levels A*β* 1-40 and A*β* 1-42 were measured by the enzyme-linked immunosorbent assay (ELISA), using the anti-rat amyloid beta peptide 1-40 or A*β* 1-42 precoated microplates (12 × 8 microwell strips).

#### 2.3.7. Determination of Serum TNF*α*

TNF*α* was measured as described by the commercial TNF*α* ELISA kit purchased from RayBiotech, USA. The TNF*α* level was determined by the enzyme-linked immunosorbent assay (ELISA) using the anti-rat TNF*α* precoated microplates (12 × 8 microwell strips).

#### 2.3.8. Determination of Acetylcholinesterase (AChE)

Activity was measured according to Ellman et al. [27].

#### 2.3.9. Nitric Oxide Assay

Nitric oxide level was estimated according to Hummel et al. [28].

#### 2.3.10. Monoamine Oxide (MAO) Assay

667 *μ*L of 500 *μ*M p-tyramine and 133 *μ*L potassium phosphate buffer pH 7.6 were added to 100 *μ*L brain, liver supernatant, or serum. The absorbance was measured at 250 nm against air after 30 s and 90 s. The activity of MAO (U/L) was calculated according to Sandler et al. [29] with the following equation:

(2) $$\begin{array}{c} \text{MAO Activity}=\frac{\Delta A\times \text{Total volume}\times 1000}{32.2\times \text{Sample volume}\times 0.5}. \end{array}$$

#### 2.3.11. Determination of Blood Retinol Binding Protein-4 (RBP4) Level

RBP4 was assayed according to the description of the commercial RBP4 ELISA kit purchased from RayBiotech, USA, using biotinylated RBP4 antibody and streptavidin-peroxidase conjugate. The absorbance was monitored on a microplate reader at a wavelength of 450 nm immediately. A standard curve was generated using the RBP4 standard concentrations on the *x*-axis against the mean absorbance at 450 nm on the *y*-axis. Unknown sample concentration was determined using the standard curve.

#### 2.3.12. Reverse Transcriptase-Polymerase Chain Reaction (RT-PCR) Analysis

Total RNA was extracted from frozen tissues using TRIzol reagent (Invitrogen). The amount and quality of RNA were assessed using a BioRad spectrophotometer and an Agilent 2100 Bioanalyzer. To examine the mRNA levels, cells were briefly washed twice with ice-cold RNase-free PBS. Two micrograms of RNA were reverse transcribed to cDNA (Ambion, Austin, TX) in 25 *μ*L of total reaction volume. Primer sequences of the analyzed genes: amyloid beta precursor protein (A*β*PP) F: GCA GAA TGG AAA ATG GGA GTC AG; R:  AAT CAC GAT GTG GGT GTG CGT C and acetylcholinesterase (AChE) F:  TTC TCC CAC ACC TGT CCT CAT C; R:  TTC ATA GAT ACC AAC ACG GTT CCC. The amplification was performed on a thermal cycler (Applied Biosystems, Foster City, CA) with different conditions for each gene. The resulting products were visualized on agarose gels. The intensity of the bands was quantified by the densitometer (Imaging Research, St. Catharines, Ontario, Canada), and resulting data were normalized by using the corresponding GAPDH (F:  TACCCCACGGCAAGTTCAATGG; R: AGGGGCGGAGATGATGATGACCC).

### 2.4. Histopathology

Sections of liver were fixed in 10% neutral buffered formalin for 48 h. Specimens were dehydrated and embedded in paraffin, sectioned, and stained with hematoxylin and eosin (H&E) for histopathological examination.

### 2.5. Molecular Docking

Berberine was docked into the crystal structures of acetylcholinesterase (AChE) (PDB code 2CMF) and HMG-CoA reductase (PDB code 1HWK). Two known drugs were also docked, including atorvastatin (statin drug, HMG-CoA reductase inhibitor) and donepezil (AChE inhibitor), and used as reference for comparison to berberine. AutoDock 3.0 [30] and MOE [31] software were used for all docking calculations. The AutoDockTools package was employed to generate the docking input files and to analyze the docking results [30]. A grid box size of 90 × 90 × 90 points with a spacing of 0.375 Å between the grid points was generated that covered almost the entire protein surface. Legends were fully flexibly docked. All nonpolar hydrogens and crystallographic water molecules were removed prior to the calculations. The docking grid was centered on the mass center of the bound TSA. In each case, 100 docked structures were generated using genetic algorithm searches. A default protocol was applied with an initial population of 50 randomly placed conformations, a maximum number of 2.5 × 105 energy evaluations, and a maximum number of 2.7 × 104 generations. A mutation rate of 0.02 and a crossover rate of 0.8 were used. Heavy atom comparison root mean square deviations (RMSD values) were calculated and initial ligand binding modes were plotted. Protein-ligand interaction plots were generated using MOE, 2012 [31].

### 2.6. Statistical Analysis

Data are reported as the mean ± S.D. One-way analysis of variance (ANOVA) was followed by Student Newman-Keuls test, which was provided by the Primer Biostatistics program (Version 5). The differences were considered statistically significant at *P* values < 0.05.

## 3. Results

In this study, we used CCL_4_ to induce NASH and to investigate if there is any accompanied neurotoxicity effect. Here, we also studied the therapeutic effects of berberine on both NASH complications and its accompanied diseases. Our measurements focused on some biochemical parameters related to oxidative stress, inflammation, lipid profile, liver function, and neurotoxicity.

### 3.1. The Effect of Berberine Treatment on NASH Biomarkers and Accompanied Dyslipidemia

Liver injury and steatosis were diagnosed by measuring the changes in the liver enzymes; aspartate aminotransferase (AST) and alanine aminotransferase (ALT) and their ratio. These enzymes' level was significantly increased in the NASH induced group compared to the control group and the berberine treated group; giving AST/ALT ratio lower than 1 (0.67) (Table 1). Furthermore, we measured the serum retinol-binding protein 4 (RBP-4) levels as an indicator marker for the severity of steatosis and NASH progress. The NASH group had a significant increase in the RBP-4 levels compared to the control and the berberine treated group (Table 1). NASH induced group showed a remarkable dyslipidemia. This was manifested by the significant increase in the blood triglycerides, LDL, VLDL, LDL/HDL ratio, and cholesterol levels. Upon berberine treatment, a significant reduction in the blood triglycerides, LDL, VLDL, LDL/HDL ratio, and cholesterol levels was observed compared to the NASH group (Table 1). Furthermore, a marked increase in the cholesterol concentration in the brain tissue in the NASH induced group was compared to the control group. Around a 54% decrease in the brain cholesterol level in the berberine-treated group was compared to the NASH induced group.

### 3.2. The Effect of Berberine on Antioxidant Capacity and Associated Inflammation

**Figure 1:**
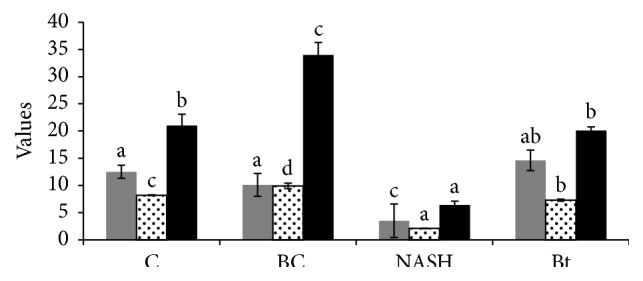
Effect of berberine treatment on the changes in brain oxidative stress factors of different rat groups. C: control group, BC: berberine control group, NASH: the CCL_4_-NASH induced group, and Bt: the CCl_4_-NASH induced group treated with berberine. The figure shows the data of GSH (black bars), SOD (dotted white bars), and TBARS (gray bars).

In the NASH induced group, the production rate of thiobarbituric acid-reactive substances (TBARS) in the serum and brain tissue samples was significantly increased in the blood samples, reflecting the increase in the lipid peroxidation. Moreover, a marked decrease in the levels of the blood endogenous antioxidant markers such as GPx, SOD, and GSH was observed in the NASH induced group (Table 2). In contrast, the berberine treated group showed a significant increase in the antioxidant system which is accompanied by a marked decrease in the TBARS. The results of the berberine treated group are similar to that of the berberine vehicle group and control group. The level of the blood xanthine oxidase also revealed 2.8-fold increase in the NASH induced group relative to the control group. This increase in the xanthine oxidase was reversed in the berberine treated group, giving value similar to that of the control group. Also, the data in the brain tissues of the NASH group revealed a marked increase in the TBARS and 1.5-fold increase in the xanthine oxidase with a significant decrease in the antioxidant markers (Table 3, Figure 1). Berberine treatment decrease the lipid peroxidation and increase the antioxidant parameters in the brain tissues (Figure 1). Our data also showed a significant increase in tumor necrosis factor (TNF*α*) which markedly decreased after berberine treatment, giving results similar to that of the control group. Moreover, the NASH induced group showed around threefold increase in the nitric oxide level which significantly decreases to value similar to that of the control group upon berberine treatment.

### 3.3. NASH Induction and Its Related Hyperglycemia and Hyperinsulinemia

The blood glucose concentration was significantly increased from 78 mg/dL in the control group to 180 mg/dL in the NASH induced group, reflecting hyperglycemia in the NASH induced group. The berberine control group showed a slight decrease in the blood glucose level compared to the control group (Table 4). Furthermore, twofold increase in the blood insulin level (20 pg/L) in the NASH induced group was compared to the control group (10.2 pg/L), suggesting hyperinsulinemia in the NASH induced group. Upon berberine treatment, the blood insulin level was returned back to a value of 10.1 pg/L which is similar to the control group. The calculated HOMA-IR was markedly decreased from 8.9 to 2.2 upon berberine treatment. In the brain tissues of NASH group a significant reduction in the glucose with around 3-fold increase in the insulin was observed. Berberine treatment reduced both the glucose and insulin levels in the brain tissues (Table 4).

### 3.4. NASH Triggers Neurotoxicity

**Figure 2:**
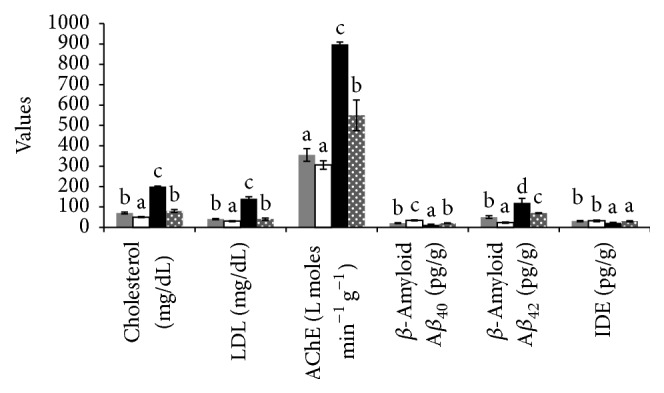
Effect of berberine treatment on serum cholesterol, LDL and brain tissue AChE, A*β*_40_, A*β*_42_, and IDE. C: control group, BC: berberine control group, NASH: the CCL_4_-NASH induced group, and Bt: the CCl_4_-NASH induced group treated with berberine. Control group (gray bars), berberine control group (white bars), NASH induced group (black bars), and NASH induced group treated with berberine (dotted gray bars).

**Figure 3:**
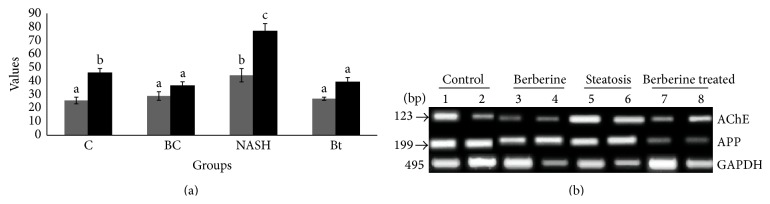
RT-PCR analysis of AChE and APP expression. The expression level of APP. (a) The relative expression of mRNA to housekeeping gene; C: control group, BC: berberine control group, NASH: the CCL_4_-NASH induced group, and Bt: the CCL_4_-NASH induced group treated with berberine. APP (gray bars) and AChE (back bars); (b) gel electrophoresis of cDNA of the two genes.

In blood and brain samples, the NASH induced group showed a significant increase in the AChE activity compared to the control group (Table 5). Moreover, around 1.5- and 2.5-fold increase in the MAO activity in the blood and brain tissues of the NASH induced group was compared to the control group, respectively. Berberine treatment markedly decreases the AChE and MAO activity in both blood and brain tissues (Table 5). A marked increase in the *β*-amyloid (A*β*_42_) and decrease in the A*β*_40_ were observed in the brain tissues of NASH induced rats. Upon berberine treatment, the *β*-amyloid (A*β*_42_) level was decreased while A*β*_40_ increased to values similar to that of the control group. Furthermore, IDE activity was decreased in the NASH induced group but it was returned back to a value similar to that of the control by berberine treatment (Table 5, Figure 2). To investigate the long-term effects of NASH on genes and proteins that can be upregulated during neurotoxicity induction, we measured amyloid-*β*-precursor protein (A*β*PP) and AChE mRNA levels by qRT-PCR analysis. Here we used glyceraldehyde-3-phosphate dehydrogenase (GAPDH) as a housekeeping gene. The RT-PCR studies demonstrated that NASH induction increased the expression of A*β*PP gene and AChE gene. In contrast, berberine treatment showed a significant decrease in the expression of both genes (Figure 3).

### 3.5. Histopathology of Liver Tissues

**Figure 4:**
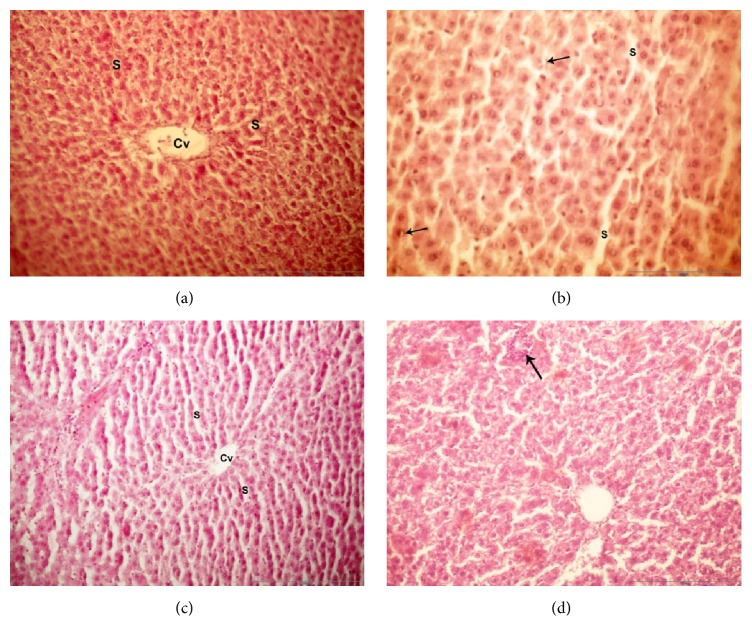
Light microscope examination of liver tissues: photomicrograph of liver sections of the different groups using (H&E 40x magnification); (a) section of the liver tissue of control group showed normal hepatic cells radiating around the central vein (Cv) and separated by sinusoids (S). (b) In the CCL_4_-NASH induced group, the liver had hepatic cells radiating around the central vein (Cv) separated by sinusoids (S) and the black arrows refer to inflammatory reaction with polymorphonuclear leukocytes surrounding hepatocytes. (c) Section of liver tissue of berberine control group showed a normal histological structure of the hepatic cords, sinusoids (S), and central vein (Cv). (d) The CCL_4_-NASH induced group treated with berberine had liver with little vacuolation of hepatocytes and central veins with RBCs (black arrow).

Histopathological studies showed that the liver of treated rats with CCL_4_ revealed a NASH pathology with ballooning, lobular inflammation typically localized in acinar zone which contained obvious fat droplets with obvious necrosis and inflammation. There were mixed patterns of macrovesicular hepatic steatosis with the conspicuous disorganization of the hepatic cord architecture with scattered foci of hepatocellular necrosis (Figure 4(b)). Berberine administration for NASH group showed disappearance of necrosis and inflammation sites and the statuses decreased from moderate to mild grade with reduced necrotic and macrovesicular structure (Figure 4(d)).

### 3.6. Molecular Docking Analysis

**Figure 5:**
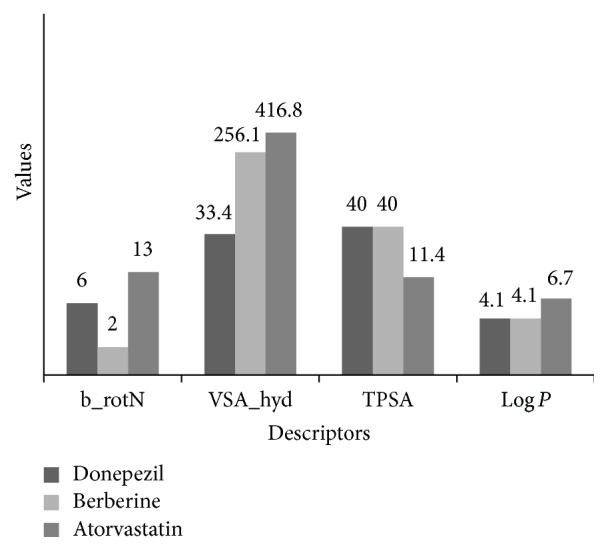
Calculated 2D compound properties of berberine compared to reference drugs; here is the reports of 2D molecular analysis of the three compounds, donepezil, berberine, and atorvastatin, showing different parameters controlling their activity. TPSA (topological polar surface area), Log *P* values (reflect the overall lipophilicity), b_rotN (number of rotatable bonds), and VSA_hyd (approximation to the sum of VDW surface of the hydrophobic atoms).

Here we used molecular docking to compare the binding of berberine and HMG-CoA reductase inhibitor (atorvastatin) to HMG-CoA reductase. Moreover, we also conducted the molecular docking analysis to compare the binding of berberine and an AChE inhibitor (donepezil) to AChE. 2D ligands molecular properties were calculated that accounted for several characteristics, including flexibility (number of rotatable bonds), polarity (total polar surface area), and hydrophobicity (VDW surface area). The comparison is reported in (Figure 5) and showed a full overview about the binding chemistry of these compounds. This analysis revealed that berberine is less flexible and more rigid in structure than the known drugs, donepezil and atorvastatin. However, it has comparable properties which are consistent with its observed activity.

#### 3.6.1. Berberine Binding Mode to AChE in Comparison with Donepezil as a Reference Drug

**Figure 6:**
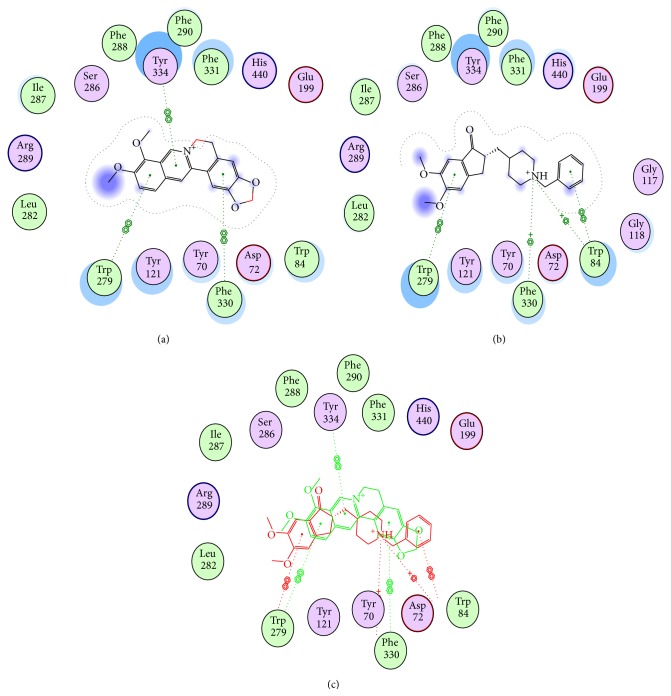
Structural models of berberine as AChEI; three plots are represented; (a) proposed binding mode of berberine in the active site of AChE; (b) docking model of reference drug donepezil; (c) comparison of putative binding modes of both berberine (red) and donepezil (green).

The structure of the AChE enzyme shows dual binding sites for inhibitors, the peripheral binding site and the catalytic binding site, PBS and CBS, respectively. Usually, all AChE inhibitors consist of two moieties representing different pharmacophores that are linked by an appropriate chain to simultaneously bind to both the peripheral and catalytic sites (PBS and CBS) of AChE. The PBS and CBS are separated by about 14 Å and located at the mouth and the bottom of the gorge of AChE, respectively. Berberine and the known drug donepezil were docked and compared (Figure 6). The isoquinoline moiety of berberine adopted an appropriate orientation to form a *π*-*π* stacking (hydrophobic aromatic) with the benzene ring of Tyr334 in PBS pocket. In addition, hydrophobic interactions were formed between the benzodioxole part of berberine and the backbone Phe330. The remaining part of berberine also interacted with Tyr121 and Phe330, resembling the reference ligand donepezil (Figures 6(a), 6(b), and 6(c)). Thus, berberine might have a binding mode similar to that of donepezil.

#### 3.6.2. Berberine Binding Mode to HMG-CoA Reductase Compared to Atorvastatin as Reference Drug

**Figure 7:**
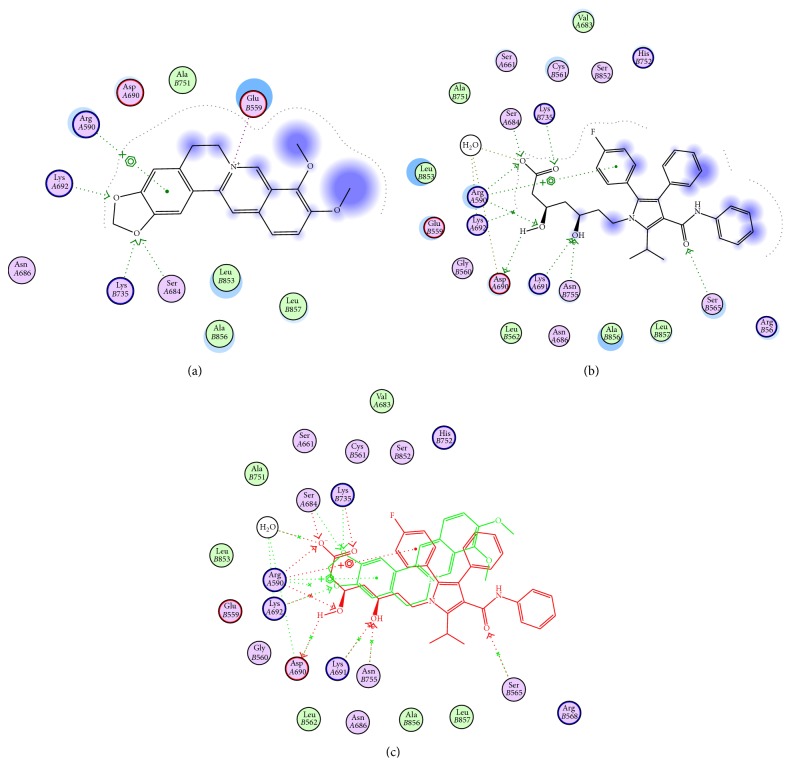
Structural models of berberine as HMG-CoA reductase inhibitor; three plots are represented, (a) proposed binding mode of berberine in the active site of HMG-CoA; (b) atorvastatin bound to crystal structure of HMG-CoA reductase; (c) comparison of putative binding modes of both berberine (red) and atorvastatin (green).

Here, we have studied the putative binding mode of berberine in the active site of HMG-CoA enzyme* via* molecular docking in comparison to atorvastatin (Figure 7). In the X-ray structure of the HMG-CoA reductase/atorvastatin complex, the ligand forms two strong hydrogen bonding interactions through an aliphatic hydroxyl group with residues Asn755 and Glu559, respectively, and additional hydrogen bonding interactions through the terminal carboxyl group and amide carbonyl with Lys735 and Ser565. Docking of berberine showed that similar mode of binding could be exhibited through the dioxole ring moiety with Ser684, Lys735, and Lys692 residues by stable hydrogen-bonding interactions. Additional aromatic hydrophobic interaction might be formed* via* the benzodioxole fragment with Arg590 amino acid (Figures 7(a), 7(b), and 7(c)). Thus, berberine might adopt a binding mode similar to atorvastatin that enhances working by the same mechanism.

## 4. Discussion

Steatohepatitis is recognized as one of the major causes of chronic liver diseases which is characterized by elevated liver enzymes. It was reported that NASH is associated with some metabolic disorders, including dyslipidemia and Alzheimer-like disease pathogenesis [16]. In our study, we tried to investigate two main aims:* first,* to speculate the mechanism by which NASH progression can trigger neurotoxicity;* second,* to investigate the therapeutic effects of berberine against NASH complications and the developed neurotoxicity.

Our study demonstrated that berberine treatment seemed to improve liver function through rebalanced lipid metabolism, attenuated inflammation, and oxidative stress. Our results are consistent with a recent study that revealed that berberine has a potent hepatoprotective effect as well as anti-inflammatory effect against NASH [11]. Furthermore, our data is consistent with what has been reported that berberine has a protective effect against CCL_4_-induced liver injury and hepatotoxicity [32]. In our study, The protective effect of berberine against liver injury in NASH was manifested by lowering the blood levels of ALT and AST. These enzymes are considered as screening tools of liver damage and inflammation. Generally the ratio AST/ALT exceeds 1 with the cirrhosis development [33]. In our study, berberine treatment lowered the AST/ALT ratio to be less than 1, suggesting its protective effect. Berberine treatment also lowered the level of the retinol binding protein-4 (RBP4) compared with the untreated group. RBP4 is the specific carrier protein of retinol in the blood and it is mainly synthesized in liver. It was shown that RBP4 is significantly elevated in sever and moderate NASH compared to mild NASH [34]. Thus it was used as a good biomarker in many liver diseases. In agreement with the previous results, berberine improved the liver histopathology and demonstrated a reduction in the inflammation and necrosis in the tissues.

In this study, berberine exerted antilipemic effect. This effect was manifested by lowering the total cholesterol, LDL, triglycerides, and LDL/HDL ratio. Our docking analysis showed that berberine might have a similar HMG-CoA reductase binding characteristic to atorvastatin (HMG-CoA reductase inhibitor). Hence, the antilipemic effect of berberine is probably due to its inhibitory activity on HMG-CoA reductase. It was reported that HMG-CoA reductase inhibitors were effective in managing hyperlipidemia and its associated diseases such as coronary artery diseases [35]. The antilipemic effect of these inhibitors was manifested through lowering the level of total cholesterol and LDL-cholesterol [35]. However, all statins have risk to cause rhabdomyolysis at high doses [36]. Hence a natural HMG-CoA reductase inhibitor or a combination between low dose of statins and a natural HMG-CoA reductase inhibitor will be a helpful alternative therapy. Recently, it has been supported that berberine has antioxidant activity [37]. Our results also demonstrated the antioxidant capacity of berberine through the reduction in the TBARS level and the increase in the antioxidant system parameters such as GSH and SOD. Taken together, the therapeutic effect of berberine against the NASH-associated hyperlipidemia which observed in our study is probably due to a combined effect of its inhibitory activity of HMG-CoA reductase and its antioxidant capacity.

Furthermore, oxidative stress is frequently stated to be a central mechanism of hepatocellular injury in NASH. Here, the oxidative stress in the brain tissue plays a significant role in neurotoxicity. Here, we observed that the increase in the oxidative stress was associated with an increase in the level of TNF*α*. It was shown that lipid accumulation is a potent cause of increased generation of oxidative stress and subsequent expression of proinflammatory cytokines such as TNF*α* and IL-6 [38]. In the literature, TNF*α* is considered as a key cytokine that has the ability to start the inflammatory cascade. Thus in our study we focused to measure this cytokine and to investigate its role in the development of neurotoxicity. It was reported that the increase in TNF*α* is associated with the pathogenesis of Alzheimer disease (AD) [39]. Here, the increased level of TNF*α* was associated with increase in the expression of* AβPP* gene in the NASH induced group. The beta amyloid peptides can play various physiological and pathological roles depending on the path of its formation. Normally, A*β* is produced from amyloid precursor protein (APP) under the action of *β* and *γ*-secretases* via* the mechanism of intramembrane proteolysis. It was reported that the oxidative stress induced by different agents increased A*β* production in cell culture [40]. The increase in the A*β*_42_ and the decrease in the A*β*_40_ were indicated in the AD pathogenesis. Taken together, the observed increase in the A*β*PP and its proteolysis product A*β*_42_ in our study is probably due to the effect of both of the increased oxidative stress and TNF*α*. Hence, the anti-inflammatory and the antioxidant activities of berberine resulted in a significant decrease in the TNF*α* and the consequent decrease in the A*β*PP and A*β*_42_ which was associated with an increase in the A*β*_40_ level. Our results supported the synergistic interaction between dyslipidemia disorders and oxidative stress in increasing the risk factors for the neurotoxicity and the onset of Alzheimer-like disease pathogenesis.

Also, our results showed that the defect in the lipid and cholesterol metabolism was associated with upregulation of the AChE and APP genes expression which may speculate their role in neuronal and synaptic impairment. Berberine treatment showed a potential inhibitory activity against AChE activity. Thus it can act as acetylcholinesterase inhibitor (AChEI), providing a novel therapeutic agent against the diseases in which a higher acetylcholinesterase activity is indicated such as Alzheimer's disease pathogenesis. Our docking analysis supports our biochemical analysis because it showed that berberine can bind to AChE in a similar manner to that of donepezil. It was shown that acetylcholine decreased the release of the proinflammatory cytokine [41]. Thus the AChE inhibitory activity of berberine will share in part in its anti-inflammatory activity. It was demonstrated that statins limit the amyloidogenic pathway* via* inhibiting the dimerization of *β*-secretase [42]. Further, statins improved dyslipidemia and inhibited the production of A*β* by disrupting secretase enzyme function and reducing neuroinflammation [43]. Thus the inhibitory activity of berberine against HMG-CoA reductase might also have a role in the improvement of Alzheimer-like pathology.

It was reported that obesity develops insulin resistance [44]. Thus the observed hyperlipidemia in our study is one of the causes of the observed hyperglycemia and hyperinsulinemia. Furthermore, many studies suggested that TNF*α* is one of the major causes of insulin resistance. It was also shown that anti-TNF*α* treatment decreases the insulin resistance in type II diabetes mellitus [45]. Having said that the observed hyperglycemia and hyperinsulinemia in our study resulted from both effects of hyperlipidemia and the high TNF*α* in the NASH induced group, thus the antilipemic and anti-inflammatory effects of berberine attenuated the hyperglycemia and hyperinsulinemia which are observed in the NASH induced group. Also, it was reported that hyperinsulinemia significantly increased the risk for Alzheimer disease [46]. Our study revealed a significant decrease for insulin-degrading enzyme (IDE) activity in the hippocampus of NASH induced rats accompanied with decreased soluble nontoxic antiamyloidgenic agent A*β*_40_. IDE cleaves small proteins such as amyloid *β*-protein (A*β*) and insulin. Thus, the amyloid accumulation in the NASH induced group is enhanced by limiting A*β* degradation* via* decreasing the insulin sensitivity which in turn competes for the IDE sites and impairs aggregated amyloids clearance.

Furthermore, the significant increase in NO in our study is considered as one of the risk factors for Alzheimer-like disease development. Our suggestion is supported by Fernandez et al. who showed that NO is one of the factors that is involved in the AD progression [47]. They suggested that NOS inhibitors will be beneficial in the AD treatment. It was reported that cytokines such as TNF*α* and interleukin-1 enhance the production of some inflammatory mediators, including NO [48]. Another study by Cordes et al. demonstrated that NO donor acts as IDE inhibitor via S-nitrosylation of the protein. This protein modification affects both the insulin and A*β* degradation by IDE [49]. Hence, NO plays a significant role in the insulin resistance and AD progression.

In our study, the observed increase in MAO activity in the NASH induced rats plays a crucial role in the AD pathogenesis. Our suggestion is supported by Yáñez and Viña who showed that dual inhibitors of MAO and AChE are effective in the AD treatment [50]. In this study, The observed reduction in the MAO-B activity mostly resulted from the inhibitory effect of berberine against MAO-B. These results are supported by Ji and Shen who showed that berberine binds MAO-A and MAO-B with theoretical *K*_*d*_ 105.2 and 66 *μ*M, respectively [51].

## 5. Conclusion

In conclusion, nonalcoholic steatohepatitis with its health complications including dyslipidemia, cholesterol impairments, oxidative stress, and upregulation of AChE with amyloid precursor protein (APP) are considered potential dangerous risk factors for neurotoxicity. Our study showed that the marked hyperlipidemia associated with NASH induction plays a crucial role in the development of the inflammation and the oxidative stress. Furthermore, our study showed that the dyslipidemia contributes to the development of other complications such as insulin resistance. All these complications, in particular, the decrease in the insulin sensitivity and the elevated TNF*α*, play a major role in the development of Alzheimer-like pathology which was manifested in our study by high AChE activity and expression, increased A*β*_42_, decreased A*β*_40_, increased NO, decreased IDE, and high MAO activity. Our promising results of alkaloid berberine treatment showed its potential therapeutic activity against the NASH and its associated diseases such as insulin resistance and Alzheimer-like pathology* via* its effect as antioxidant, anti-inflammatory, an AChE inhibitor and HMG-CoA reductase inhibitor. Finally, our study enhances the recommendation of berberine as potential natural therapeutic agent for treatment of hepatic diseases, insulin resistance, and Alzheimer-like disease pathogenesis.

## Figures and Tables

**Table 1:** Mean levels of blood liver function tests and lipid profile in control, NASH, and berberine treated groups.

Groups	AST (U/L)	ALT (U/L)	AST/ALT	RBP-4 (pg/L)	TG mg/dL	Cholesterol mg/dL	LDL mg/dL	VLDL mg/dL	LDL/HDL	Brain cholesterol (mg/g)
Control	12 ± 1.2^a^	10 ± 0.4^a^	1.2	200.5 ± 19^a^	56 ± 7.5^a^	70 ± 4.5^b^	40 ± 2.3^b^	11 ± 1.22^b^	2.67	90 ± 10^a^
Berberine control	13 ± 2.1^a^	8.5 ± 0.32^a^	1.53	190 ± 20^a^	40 ± 2.3^a^	50 ± 2.3^a^	30 ± 2.1^a^	8 ± 2.1^a^	2.5	98 ± 12^a^
NASH	30 ± 3.2^b^	45 ± 4.2^b^	0.67	600 ± 76^c^	180 ± 23^c^	200 ± 23^c^	140 ± 10^c^	36 ± 3.1^d^	5.8	238.7 ± 34.9^c^
Berberine treatment	15 ± 2.5^a^	12 ± 4.2^a^	1.25	230 ± 8.5^ab^	70 ± 11^ab^	80 ± 6.7^b^	40 ± 5.5^b^	14 ± 2.1^c^	1.54	130 ± 9.9^b^

Values are reported as means ± SD of rat groups (10 rats each) with different degrees of significance at *P* < 0.05.

^a^Lowest value of significance.

^d^Highest value of significance.

If two groups have the same letters, it means no significant difference is detected.

**Table 2:** Mean levels of serum oxidative stress and proinflammatory parameters in control, NASH, and berberine treated groups.

Groups	TBARS nmol/mL	XO (*µ*M/hour)	GPx (IU)	SOD (IU)	GSH (mg/dL)	TNF-alpha (pg/L)	NO (nmole/mL)
Control	2.3 ± 0.2^b^	13.4 ± 1.6^a^	7.2 ± 0.5^c^	3.4 ± 0.2^b^	3.2 ± 0.3^b^	87 ± 12^a^	9.3 ± 3.1^b^
Berberine control	1.5 ± 0.1^a^	12.1 ± 2.2^a^	9.1 ± 0.5^d^	4.1 ± 0.2^c^	4 ± 0.1^c^	76 ± 9.6^a^	5 ± 0.9^a^
NASH group	10 ± 1.2^d^	37.2 ± 5.2^b^	4.2 ± 0.2^a^	2.1 ± 0.1^a^	1.4 ± 0.2^a^	500 ± 45^c^	30 ± 7.2^c^
Berberine treatment	5 ± 0.9^c^	13.2 ± 4.2^a^	6.1 ± 0.2^b^	3.1 ± 0.5^b^	3.2 ± 0.2^b^	150 ± 30^b^	10.3 ± 2.3^b^

Values are reported as means ± SD of rat groups with different degrees of significance at *P* < 0.05.

^a^Lowest value of significance.

^d^Highest value of significance.

If two groups have the same letters, it means no significant difference is detected.

**Table 3:** Mean levels of brain oxidative stress and proinflammatory parameters in control, NASH, and berberine treated groups.

Groups	TBARS (nM/mL)	XO (*µ*M/hour)	GPx (IU)	SOD (IU)	GSH (mg/dL)	TNF-alpha (pg/L)	NO (nmole/mL)
Control	12.5 ± 1.2^a^	8.2 ± 0.9^a^	0.02 ± 0.001^b^	8.2 ± 0.07^c^	21 ± 2.1^b^	60.5 ± 3.5^b^	5.6 ± 0.5^a^
Berberine control	10.1 ± 2.1^a^	7.5 ± 0.4^a^	0.02 ± 0.001^b^	9.9 ± 0.5^d^	34 ± 2.3^c^	50.4 ± 6.2^a^	4.3 ± 0.6^a^
NASH group	30.5 ± 3.1^c^	12.3 ± 0.6^c^	0.01 ± 0.007^a^	2.1 ± 0.04^a^	6.4 ± 0.7^a^	205 ± 12.5^c^	54.1 ± 2^d^
Berberine treatment	14.6 ± 1.9^ab^	10.1 ± 0.9^b^	0.019 ± 0.0001^b^	7.3 ± 0.2^b^	20.1 ± 0.65^b^	89 ± 10.1^c^	11.5 ± 1^c^

Values are reported as means ± SD of rat groups with different degrees of significance at *P* < 0.05.

^a^Lowest value of significance.

^d^Highest value of significance.

If two groups have the same letters, it means no significant difference is detected.

**Table 4:** Mean levels of insulin and glucose levels in the blood and brain tissues in control, NASH, and berberine treated groups.

Groups	Insulin^A^ (pg/L)	Glucose^A^ (mg/dL)	HOMA-IR^A^	Insulin^B^ (pg/l0 g)	Glucose^B^ (mg/10 g)
Control	10.2 ± 1.1^a^	78 ± 2.9^b^	1.9 ± 0.007^b^	3.5 ± 0.2^a^	5.3 ± 0.4^b^
Berberine control	9.9 ± 0.2^a^	60 ± 3.2^a^	1.5 ± 0.009^a^	3.2 ± 0.2^a^	5.1 ± 0.1^b^
NASH group	20 ± 1.9^b^	180 ± 12^d^	8.9 ± 0.1^d^	10.2 ± 0.9^c^	1.2 ± 0.2^a^
Berberine treatment	10.1 ± 0.7^a^	90 ± 8.4^c^	2.2 ± 0.004^c^	4.9 ± 0.1^b^	5.3 ± 0.5^b^

The superscript letter A: the parameters that were measured in the blood.

The superscript letter B: the parameters that were measured in the brain tissues.

Values are reported as means ± SD of rat groups with different degrees of significance at *P* < 0.05.

^a^Lowest value of significance.

^d^Highest value of significance.

If two groups have the same letters, it means no significant difference is detected.

**Table 5:** Mean levels of brain parameters in control, NASH, and berberine treated groups.

Groups	AChE^A^ (liter moles min^−1^ mL^−1^)	MAO^A^ (IU)	*β*-amyloid^B^ A*β*_40_ (pmol/L)	AChE^B^ (liter moles min^−1^ g^−1^)	MAO^B^ (IU/mg)	*β*-amyloid^B^ A*β*_40_ (pmol/L)	IDE^B^
Control	43 ± 2.1^b^	8.2 ± 1.1^b^	5.6 ± 0.8^b^	355 ± 31.1^a^	3.1 ± 0.3^a^	50 ± 6.5^b^	30.6 ± 2.53^b^
Berberine control	36 ± 2.1^a^	4.3 ± 0.9^a^	6 ± 0.1^b^	306 ± 21^a^	3.2 ± 0.4^a^	23 ± 3.4^a^	32 ± 3.94^b^
NASH group	98 ± 12^d^	12 ± 3^c^	2.1 ± 0.1^a^	898 ± 12^c^	8.1 ± 0.9^c^	120 ± 22^d^	22 ± 0.9^a^
Berberine treatment	55 ± 7.5^c^	9 ± 1.1^b^	6.3 ± 0.9^b^	550 ± 75^b^	4.3 ± 0.5^b^	70 ± 1.9^c^	29.3 ± 2.7^a^

The superscript letter A: the parameters that were measured in the blood.

The superscript letter B: the parameters that were measured in the brain tissues.

Values are reported as means ± SD of rat groups with different degrees of significance at *P* < 0.05.

^a^Lowest value of significance.

^d^Highest value of significance.

If two groups have the same letters, it means no significant difference is detected.

## References

[1] Marchesini G., Bugianesi E., Forlani G. (2003). Nonalcoholic fatty liver, steatohepatitis, and the metabolic syndrome. *Hepatology*.

[2] Xiong H., Callaghan D., Jones A. (2008). Cholesterol retention in Alzheimer's brain is responsible for high beta- and gamma-secretase activities and Abeta production. *Neurobiology of Disease*.

[3] Di Paolo G., Kim T.-W. (2011). Linking lipids to Alzheimer's disease: cholesterol and beyond. *Nature Reviews Neuroscience*.

[4] Ghareeb D., Al Sayed A. N., Rashidy F. H., Hussein H. M., Asmaa N. A. (2010). Efficacy of natural extracts of *Ginkgo biloba* and berberry and a synthetic derivative of genistein (ipriflavone), as acetylcholinesterase inhibitors, comparative study with Aricept effect. *Journal of Biochemistry and Biotechnology*.

[5] Buxbaum J. D., Cullen E. I., Friedhoff L. T. (2002). Pharmacological concentrations of the HMG-CoA reductase inhibitor lovastatin decrease the formation of the Alzheimer beta-amyloid peptide in vitro and in patients. *Frontiers in Bioscience*.

[6] Inglis F. (2002). The tolerability and safety of cholinesterase inhibitors in the treatment of dementia. *International Journal of Clinical Practice, Supplement*.

[7] Farris W., Mansourian S., Chang Y. (2003). Insulin-degrading enzyme regulates the levels of insulin, amyloid *β*-protein, and the *β*-amyloid precursor protein intracellular domain in vivo. *Proceedings of the National Academy of Sciences of the United States of America*.

[8] Birdsall T. C. (1997). Berberine: therapeutic potential of an alkaloid found in several medicinal plants. *Alternative Medicine Review*.

[9] Kong W.-J., Wei J., Zuo Z.-Y. (2008). Combination of simvastatin with berberine improves the lipid-lowering efficacy. *Metabolism: Clinical and Experimental*.

[10] Kong W. J., Wei J., Abidi P. (2004). Berberine is a novel cholesterol-lowering drug working through a unique mechanism distinct from statins. *Nature Medicine*.

[11] Xiao H.-B., Sun Z.-L., Zhang H.-B., Zhang D.-S. (2012). Berberine inhibits dyslipidemia in C57BL/6 mice with lipopolysaccharide induced inflammation. *Pharmacological Reports*.

[12] Wang N., Feng Y., Cheung F. (2012). A comparative study on the hepatoprotective action of bear bile and coptidis rhizoma aqueous extract on experimental liver fibrosis in rats. *BMC Complementary and Alternative Medicine*.

[13] Huang L., Luo Z., He F., Shi A., Qin F., Li X. (2010). Berberine derivatives, with substituted amino groups linked at the 9-position, as inhibitors of acetylcholinesterase/butyrylcholinesterase. *Bioorganic and Medicinal Chemistry Letters*.

[14] Zhu F., Wu F., Ma Y. (2011). Decrease in the production of *β*-amyloid by berberine inhibition of the expression of *β*-secretase in HEK293 cells. *BMC Neuroscience*.

[15] Abd El-Wahab A. E., Ghareeb D. A., Sarhan E. E. M., Abu-Serie M. M., El Demellawy M. A. (2013). In vitro biological assessment of berberis vulgaris and its active constituent, berberine: antioxidants, anti-acetylcholinesterase, anti-diabetic and anticancer effects. *BMC Complementary and Alternative Medicine*.

[16] Ghareeb D. A., Hafez H. S., Hussien H. M., Kabapy N. F. (2011). Non-alcoholic fatty liver induces insulin resistance and metabolic disorders with development of brain damage and dysfunction. *Metabolic Brain Disease*.

[17] Moroz L. L., Edwards J. R., Puthanveettil S. V. (2006). Neuronal transcriptome of *Aplysia*: neuronal compartments and circuitry. *Cell*.

[18] Wills E. D. (1965). Mechanisms of lipid peroxide formation in tissues Role of metals and haematin proteins in the catalysis of the oxidation of unsaturated fatty acids. *Biochimica et Biophysica Acta—Lipids and Lipid Metabolism*.

[19] Paglia D. E., Valentine W. N. (1967). Studies on the quantitative and qualitative characterization of erythrocyte glutathione peroxidase. *The Journal of Laboratory and Clinical Medicine*.

[20] Jollow D. J., Mitchell J. R., Zampaglione N., Gillette J. R. (1974). Bromobenzene induced liver necrosis. Protective role of glutathione and evidence for 3,4 bromobenzene oxide as the hepatotoxic metabolite. *Pharmacology*.

[21] Marklund S., Marklund G. (1974). Involvement of the superoxide anion radical in the autoxidation of pyrogallol and a convenient assay for superoxide dismutase. *European Journal of Biochemistry*.

[22] Fossati P., Prencipe L. (1982). Serum triglycerides determined colorimetrically with an enzyme that produces hydrogen peroxide. *Clinical Chemistry*.

[23] Watson D. (1960). A simple method for the determination of serum cholesterol. *Clinica Chimica Acta*.

[24] Friedewald W. T., Levy R. I., Fredrickson D. S. (1972). Estimation of the concentration of low-density lipoprotein cholesterol in plasma, without use of the preparative ultracentrifuge. *Clinical Chemistry*.

[25] Lopes-Virella M. F., Stone P., Ellis S., Colwell J. A. (1977). Cholesterol determination in HDL separated by three different methods. *Clinical Chemistry*.

[26] Reitman S., Frankel S. (1957). Glutamic-pyruvate transaminase assay by colorimetric method. *American Journal of Clinical Pathology*.

[27] Ellman G. L., Courtney K. D., Andres V., Featherstone R. M. (1961). A new and rapid colorimetric determination of acetylcholinesterase activity. *Biochemical Pharmacology*.

[28] Hummel S. G., Fischer A. J., Martin S. M., Schafer F. Q., Buettner G. R. (2006). Nitric oxide as a cellular antioxidant: a little goes a long way. *Free Radical Biology and Medicine*.

[29] Sandler M., Reveley M. A., Glover V. (1981). Human platelet monoamine oxidase activity in health and disease: a review. *Journal of Clinical Pathology*.

[30] Morris G. M., Goodsell D. S., Halliday R. S. (1998). Automated docking using a Lamarckian genetic algorithm and an empirical binding free energy function. *Journal of Computational Chemistry*.

[31] http://www.chemcomp.com/.

[32] Janbaz K. H., Gilani A. H. (2000). Studies on preventive and curative effects of berberine on chemical-induced hepatotoxicity in rodents. *Fitoterapia*.

[33] Angulo P., Keach J. C., Batts K. P., Lindor K. D. (1999). Independent predictors of liver fibrosis in patients with nonalcoholic steatohepatitis. *Hepatology*.

[34] Terra X., Auguet T., Broch M. (2013). Retinol binding protein-4 circulating levels were higher in nonalcoholic fatty liver disease vs. Histologically normal liver from morbidly obese women. *Obesity*.

[35] Lopez L. M. (2002). Managing hyperlipidemia: current and future roles of HMG-CoA reductase inhibitors. *American Journal of Health-System Pharmacy*.

[36] Thompson P. D., Clarkson P., Karas R. H. (2003). Statin-associated myopathy. *The Journal of the American Medical Association*.

[37] Li Z., Geng Y.-N., Jiang J.-D., Kong W.-J. (2014). Antioxidant and anti-inflammatory activities of Berberine in the treatment of diabetes mellitus. *Evidence-Based Complementary and Alternative Medicine*.

[38] Reddy J. K., Rao M. S. (2006). Lipid metabolism and liver inflammation. II. Fatty liver disease and fatty acid oxidation. *The American Journal of Physiology—Gastrointestinal and Liver Physiology*.

[39] Sharma V., Deshmukh R. (2012). Tumor necrosis factor and Alzheimer's disease: a cause and consequence relationship. *Bulletin of Clinical Psychopharmacology*.

[40] Wolfe M. S., Kopan R. (2004). Intramembrane proteolysis: theme and variations. *Science*.

[41] Uysal K. T., Wiesbrock S. M., Marino M. W., Hotamisligil G. S. (1997). Protection from obesity-induced insulin resistance in mice lacking TNF-*α* function. *Nature*.

[42] Parsons R. B., Price G. C., Farrant J. K., Subramaniam D., Adeagbo-Sheikh J., Austen B. M. (2006). Statins inhibit the dimerization of *β*-secretase via both isoprenoid- and cholesterol-mediated mechanisms. *Biochemical Journal*.

[43] Silva T., Teixeira J., Remião F., Borges F. (2013). Alzheimer's disease, cholesterol, and statins: the junctions of important metabolic pathways. *Angewandte Chemie: International Edition*.

[44] Qatanani M., Lazar M. A. (2007). Mechanisms of obesity-associated insulin resistance: many choices on the menu. *Genes & Development*.

[45] Cheung A. T., Ree D., Kolls J. K., Fuselier J., Coy D. H., Bryer-Ash M. (1998). An in vivo model for elucidation of the mechanism of tumor necrosis factor-*α* (TNF-*α*)-induced insulin resistance: evidence for differential regulation of insulin signaling by TNF-*α*. *Endocrinology*.

[46] Schrijvers E. M. C., Witteman J. C. M., Sijbrands E. J. G., Hofman A., Koudstaal P. J., Breteler M. M. B. (2010). Insulin metabolism and the risk of Alzheimer disease: the Rotterdam Study. *Neurology*.

[47] Fernandez A. P., Pozo-Rodrigalvarez A., Serrano J., Martinez-Murillo R. (2010). Nitric oxide: target for therapeutic strategies in Alzheimer's disease. *Current Pharmaceutical Design*.

[48] Goldring M. B., Berenbaum F. (2004). The regulation of chondrocyte function by proinflammatory mediators: prostaglandins and nitric oxide. *Clinical Orthopaedics and Related Research*.

[49] Cordes C. M., Bennett R. G., Siford G. L., Hamel F. G. (2009). Nitric oxide inhibits insulin-degrading enzyme activity and function through *S*-nitrosylation. *Biochemical Pharmacology*.

[50] Yáñez M., Viña D. (2013). Dual inhibitors of monoamine oxidase and cholinesterase for the treatment of Alzheimer disease. *Current Topics in Medicinal Chemistry*.

[51] Ji H.-F., Shen L. (2012). Molecular basis of inhibitory activities of berberine against pathogenic enzymes in Alzheimer's disease. *The Scientific World Journal*.

